# Antioxidant and anticholinesterase investigations of *Rumex hastatus* D. Don: potential effectiveness in oxidative stress and neurological disorders

**DOI:** 10.1186/s40659-015-0010-2

**Published:** 2015-03-26

**Authors:** Sajjad Ahmad, Farhat Ullah, Muhammad Ayaz, Abdul Sadiq, Muhammad Imran

**Affiliations:** Department of Pharmacy, University of Malakand, Chakdara, Dir Pakistan

**Keywords:** Ethnomedicinal, Acetylcholinesterase, Butyrycholinesterase, DPPH, ABTS, H_2_O_2_

## Abstract

**Background:**

*Rumex* species are traditionally used for the treatment of neurological disorders including headache, migraine, depression, paralysis etc. Several species have been scientifically validated for antioxidant and anticholinestrase potentials. This study aims to investigate *Rumex hastatus* D. Don crude methanolic extract, subsequent fractions, saponins and flavonoids for acetylcholinestrase, butyrylcholinestrase inhibition and diverse antioxidant activities to validate its folkloric uses in neurological disorders. *Rumex hastatus* crude methanolic extract (Rh. Cr), subsequent fractions; n-hexane (Rh. Hex), chloroform (Rh. Chf), ethyl acetate (Rh. EtAc), aqueous fraction (Rh. Aq), crude saponins (Rh. Sp) and flavonoids (Rh. Fl) were investigated against acetylcholinesterase (AChE) and butyrylcholinesterase (BChE) at various concentrations (125, 250, 500, 1000 μg/mL) using Ellman’s spectrophotometric analysis. Antioxidant potentials of Rh. Sp and Rh. Fl were evaluated using DPPH, H_2_O_2_ and ABTS free radical scavenging assays at 62.5, 125, 250, 500, 1000 μg/mL.

**Results:**

All the test samples showed concentration dependent cholinesterase inhibition and radicals scavenging activity. The AChE inhibition potential of Rh. Sp and Rh. Fl were most prominent i.e., 81.67 ± 0.88 and 91.62 ± 1.67 at highest concentration with IC_50_ 135 and 20 μg/mL respectively. All the subsequent fractions exhibited moderate to high AChE inhibition i.e., Rh. Cr, Rh. Hex, Rh. Chf, Rh. EtAc and Rh. Aq showed IC_50_ 218, 1420, 75, 115 and 1210 μg/mL respectively. Similarly, against BChE various plant extracts i.e., Rh. Sp, Rh. Fl, Rh. Cr, Rh. Hex, Rh. Chf, Rh. EtAc and Rh. Aq resulted IC_50_ 165, 175, 265, 890, 92, 115 and 220 μg/mL respectively. In DPPH free radical scavenging assay, Rh. Sp and Rh. Fl showed comparable results with the positive control i.e., 63.34 ± 0.98 and 76.93 ± 1.13% scavenging at 1 mg/mL concentration (IC_50_ 312 and 104 μg/mL) respectively. The percent ABTS radical scavenging potential exhibited by Rh. Sp and Rh. Fl (1000 μg/mL) were 82.58 ± 0.52 and 88.25 ± 0.67 with IC_50_ 18 and 9 μg/mL respectively. Similarly in H_2_O_2_ scavenging assay, the Rh. Sp and Rh. Fl exhibited IC_50_ 175 and 275 μg/mL respectively.

**Conclusion:**

The strong anticholinesterase and antioxidant activities of Rh. Sp, Rh. Fl and various fractions of *R. hastatus* support the purported ethnomedicinal uses and recommend *R. hastatus* as a possible remedy for the treatment of AD and neurodegenerative disorders.

## Background

According to WHO report 2001, round the world 25% individuals suffer from mental/neurological disorders in their life time [1]. The most common neurological disorders are epilepsy, headache, migraine, susto (fright), madness, numbness, insomnia, stress, depression, alzeimer’s disease, parkinson’s disease, anxiety etc [2,3]. In developed countries the treatment of these diseases is achieved by proper preventative measures, rehabilitation and improved monitoring of patients. But in developing and underdeveloped countries, the patients suffering from different diseases primarily rely on traditional practitioners and herbal medicine. The traditional use of herbal medicine is as old as the history of man. Even today the herbal medicine is utilized in sizable portion of world’s population. As far as the use of traditional medicine for the neurological diseases are concerned, the scientific validation of various medicinal plants supports their traditional uses and have led to development of novel drugs [4,5]. Large numbers of natural bioactive compounds have been derived from various plants species based on their traditional knowledge. For example the Ginkgo biloba was traditionally perceived as memory enhancer and was considered as anti-ageing which was scientifically verified but later on it was also confirmed that it is effective in the treatment of mild to moderate alzeimer’s disease [6-8]. Similarly galanthamine a drug used against AD that is derived from *Galanthus nivalis* is also the outcome of traditional knowledge. It has been reported in detail that Galanthamine basically increases acetylcholine level in synapsis by inhibiting the acetylcholinesterase responsible for the breakdown of acetylcholine. AD occurs as a result of decreased cholinergic transmission, increased oxidative stress, increased inflammatory condition, increased β-amyloid formation, decreased nerve growth factors etc [9]. But for the treatment of AD only cholinesterase inhibitors are considered primarily. The other factors responsible for AD are not emphasized as the cholinergic transmission, yet they are having ample importance.

The ethnomedicinal uses of flora make it easy to know the latent potentials of a specific plant specie. *R. hastatus*, one of the members of polygonaceae has wide variety of traditional uses, for examples, it is commonly used as laxative, alterative, tonic, in rheumatism [10], in skin diseases, piles, bilious complaints and lungs bleeding [11]. The juice of *R. hastatus* is used to treat blood pressure [12], in tonsillitis and sore throat [13], as flavoring agent, carminative and diuretic [14]. Similarly it has also been reported to be used traditionally against giddiness and insanity [15]. As far as other species of Rumex are concerned, majority of them are traditionally being used for various neurological disorders i.e., *R. acetosa* is traditionally used in paralysis [16], *R. abyssinicus* is used for the treatment of migraine, headache, rabies [17], *R. tuberosus* is used as tension regulator [18] and *R. nepalensis, R. maderensis* are also used in headache [19,20]. Moreover the R. patientia has been scientifically confirmed to improve memory and passive avoidance learning [21]. Antioxidant activities of several species of *Rumex* have been reported while the anticholinesterase activities are still to be scientifically confirmed [22,23]. Almost all the species of a specific genus resembles considerably due to genome similarity among the species of the same genus [24]. As discussed earlier, the species of *Rumex* are traditionally being used in paralysis, headache and other CNS disorders so it provide an ethnomedicinal evidence for the presence of cholinergic transmission enhancing principles in this genus. Based on the previous literature and ethnomedicinal uses, this investigational study is designed to uncover the anticholinesterase and antioxidant potentials of various samples of *R. hastatus* and to provide a bridge between the traditional and scientific knowledge.

## Results

### Phytochemical screening and extraction yields

The preliminary phytochemical screening of *R. hastatus* confirmed the presence of alkaloids, saponins, anthraquinone glycoside, tannins and flavonoids. The extraction yield of Rh. Sp and Rh. Fl of *R. hastatus* was 6.5 and 8% respectively.

### Acetylcholinesterase inhibition assay

The most prominent AChE inhibition of various samples of *R. hastatus* was recorded for Rh. Sp and Rh. Fl. The percent AChE inhibition observed for Rh. Sp were 81.67 ± 0.88, 72.87 ± 1.27, 60.95 ± 2.01 and 49.08 ± 1.04 at the concentration of 1000, 500, 250 and 125 μg/mL respectively with IC_50_135 μg/mL, while the Rh. Fl displayed 91.62 ± 1.67, 84.76 ± 0.61, 81.03 ± 0.86 and 67.70 ± 0.92% AChE inhibition at afore mentioned concentrations with IC_50_ 20 μg/mL. Among the resultant fractions the Rh. Chf revealed good results as shown in Table 1. All the fractions showed concentration dependent activity. The IC_50_ calculated for Rh. Cr, Rh. Hex, Rh. Chf, Rh. EtAc and Rh. Aq were 218, 1420, 75, 115 and 1210 μg/mL respectively, in which Rh. Chf was observed as most potent.

**Table 1:** **Percent AChE and BChE inhibition potential of various samples of** ***Rumex hastatus***

**Samples**	**Concentrations (μg/mL)**	**Percent**	**AChEI**	**Percent**	**BChEI**
		**AChEI**	**IC**_**50**_	**BChEI**	**IC**_**50**_
		**(mean ± S.E.M)**	**(μg/mL)**	**(mean ± S.E.M)**	**(μg/mL)**
Rh.Cr	1000	65.33 ± 0.49^***^		69.58 ± 1.12^***^	
	500	59.03 ± 0.23^***^	218	63.65 ± 1.34^***^	265
	250	52.03 ± 0.48^***^		49.31 ± 2.15^***^	
	125	39.00 ± 0.58^***^		41.03 ± 0.48^***^	
Rh. Hex	1000	42.67 ± 0.89^***^		51.44 ± 0.58^***^	
	500	39.00 ± 1.15^***^	1420	46.90 ± 0.96^***^	890
	250	38.00 ± 0.00^***^		39.28 ± 2.19^***^	
	125	29.03 ± 0.23^***^		32.70 ± 1.60^***^	
Rh. Chf	1000	76.70 ± 1.60^***^		75.76 ± 0.71^***^	
	500	71.73 ± 0.78^***^	75	65.65 ± 1.32^***^	92
	250	68.70 ± 1.60^***^		61.23 ± 1.83^***^	
	125	52.90 ± 0.52^***^		53.13 ± 0.20^***^	
Rh. EtAc	1000	77.23 ± 0.40^***^		74.56 ± 1.06^***^	
	500	69.90 ± 0.43^***^	115	66.42 ± 0.43^***^	115
	250	55.90 ± 0.20^***^		59.42 ± 0.46^***^	
	125	51.13 ± 0.20^***^		51.87 ± 1.27^***^	
Rh.Aq	1000	46.20 ± 0.23^***^		61.03 ± 0.35^***^	
	500	38.80 ± 0.37^***^	1210	52.08 ± 0.47^***^	220
	250	37.90 ± 0.48^***^		51.91 ± 0.88^***^	
	125	31.74 ± 0.68^***^		44.90 ± 0.96^***^	
Rh.Sp	1000	81.67 ± 0.88^***^		77.52 ± 3.28^***^	
	500	72.87 ± 1.27^***^	135	63.93 ± 0.67^***^	165
	250	60.95 ± 2.01^***^		61.83 ± 1.21^***^	
	125	49.08 ± 1.04^***^		42.22 ± 1.28^***^	
Rh.Fl	1000	91.62 ± 1.67^**^		79.16 ± 0.86^***^	
	500	84.76 ± 0.61^***^	20	77.98 ± 0.72^***^	175
	250	81.03 ± 0.86 ^ns^		59.65 ± 0.98^***^	
	125	67.70 ± 0.92 ^ns^		41.93 ± 1.01^***^	
Gal	1000	96.00 ± 0.30		88.61 ± 0.43	
	500	91.26 ± 1.27	20	83.25 ± 1.40	47
	250	82.91 ± 1.30		74.03 ± 0.86	
	125	69.91 ± 0.89		61.22 ± 1.28	

Data is represented as mean ± S.E.M; n = 3, * represent level of significance like; * = P < 0.05, ** = P < 0.01, *** = P < 0.001.

Gal : Galanthamine.

### Butyrylcholinesterase inhibition assay

In BChE inhibition assay, Rh. Fl and Rh. Sp excelled among all the test samples. The percent BChE inhibition calculated for Rh. Sp were 77.52 ± 3.28, 63.93 ± 0.67, 61.83 ± 1.21 and 42.22 ± 1.28 at 1000, 500, 250 and 125 μg/mL respectively with IC_50_ 165 μg/mL. Rh. Fl displayed 79.16 ± 0.86, 77.98 ± 0.72, 59.65 ± 0.98 and 41.93 ± 1.01% BChE inhibition at afore mentioned concentrations with IC_50_ 175 μg/mL. Similarly all the fractions showed moderate to high activity and the IC_50_ calculated for Rh. Cr, Rh. Hex, Rh. Chf, Rh. EtAc and Rh. Aq were 265, 890, 92, 115 and 220 μg/mL respectively against BChE as shown in the Table 1. Briefly the BChE inhibition observed for each plant samples were dose dependent and the results of Rh. Fl and Rh. Sp were almost comparable with the positive control.

### DPPH radical scavenging effect

The antioxidant activity of Rh. Sp and Rh. Fl against DPPH free radicals displayed a dose dependent response. The DPPH scavenging potential of Rh. Fl was comparable with ascorbic acid while the Rh. Sp displayed a moderate antioxidant activity in comparison with the positive control. The percent radical scavenging potentials of Rh. Sp were 63.34 ± 0.98, 56.32 ± 1.06, 48.05 ± 0.75, 44.70 ± 1.25 and 38.74 ± 0.68 at the concentrations of 1000, 500, 250, 125 and 62.5 μg/mL respectively with IC_50_ 312 μg/mL. The Rh. Fl demonstrated 76.93 ± 1.13, 64.74 ± 1.29, 61.42 ± 0.57, 52.34 ± 1.01 and 46.73 ± 0.78% radicals scavenging potentials at 1000, 500, 250, 125 and 62.5 μg/mL respectively with IC_50_ 104 μg/mL as shown in the Table 2.

**Table 2:** **Percent DPPH radical scavenging potential of saponins and flavonoids of** ***Rumex hastatus*** **at various concentrations**

**Samples**	**Concentrations (μg/mL)**	**Percent inhibition (mean ± S.E.M)**	**IC**_**50**_ **(μg/mL)**
Rh. Sp	1000	63.34 ± 0.98***	
	500	56.32 ± 1.06***	312
	250	48.05 ± 0.75***	
	125	44.70 ± 1.25***	
	62.5	38.74 ± 0.68***	
Rh.Fl	1000	76.93 ± 1.13^ns^	
	500	64.74 ± 1.29^ns^	104
	250	61.42 ± 0.57^ns^	
	125	52.34 ± 1.01^ns^	
	62.5	46.73 ± 0.78^ns^	
A.A	1000	77.17 ± 0.72	
	500	67.65 ± 1.47	95
	250	62.85 ± 0.97	
	125	53.37 ± 1.65	
	62.5	47.87 ± 0.26	

Data is represented as mean ± S.E.M; n = 3. * represent level of significance like; * = P < 0.05, ** = P < 0.01, *** = P < 0.001.

AA: Ascorbic acid.

### H_2_O_2_ scavenging effect

**Figure 1:**
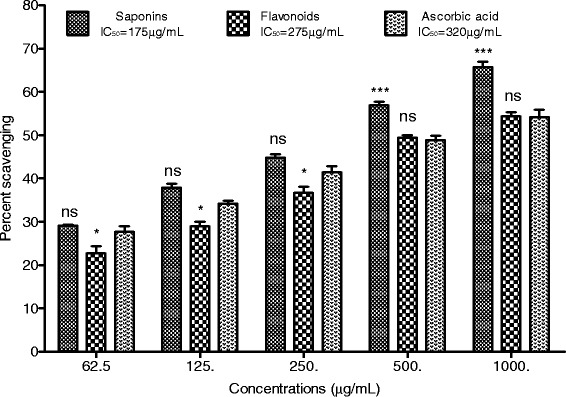
**Percent H**_**2**_ **O**_**2**_ **scavenging effect of flavonoids and saponins of** ***Rumex hastatus*** **along with ascorbic acid at various concentrations.** Data is represented as mean ± S.E.M.

The antioxidant activity of *R. hastatus* against hydrogen peroxide revealed good results for Rh. Sp. The Rh. Fl demonstrated good antioxidant activity with IC_50_ 275 μg/mL, which was comparable with the positive control. The percent hydrogen peroxide scavenging potentials exhibited by Rh. Sp were 29.13 ± 0.20, 37.85 ± 0.97, 44.82 ± 0.82, 56.90 ± 0.78 and 65.70 ± 1.25 at the concentrations of 62.5, 125, 250, 500 and 1000 μg/mL respectively with the IC_50_ of 175 μg/mL. At the highest concentration, i.e., 1000 μg/mL the Rh. Fl and positive control exhibited 54.34 ± 1.01 and 54.17 ± 1.69% inhibitions respectively while the Rh. Sp demonstrated 65.70 ± 1.25% antioxidant potentials as shown in Figure 1.

### ABTS radical scavenging effect

**Figure 2:**
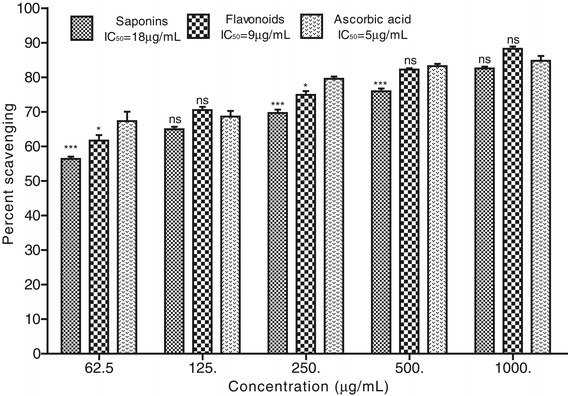
**Percent ABTS radical scavenging effect of flavonoids and saponins of** ***Rumex hastatus*** **along with ascorbic acid at various concentrations.** Data is represented as mean ± S.E.M.

The ABTS radical scavenging activity of Rh. Sp and Rh. Fl of *R. hastatus* was more prominent in comparison to other antioxidant assays. Both the plant samples exhibited very low IC_50_ values in this activity. The Rh. Fl displayed 88.25 ± 0.67, 82.23 ± 0.39, 74.92 ± 1.08, 70.53 ± 0.90 and 61.70 ± 1.60% ABTS radical scavenging potential at the concentrations of 1000, 500, 250, 125 and 62.5 μg/mL respectively with the IC_50_ of 9 μg/mL. The Rh. Sp showed the IC_50_ value of 18 μg/mL, which was larger (less potent) than those of Rh. Fl (IC_50_ = 9 μg/mL) and positive control (IC_50_ = 5 μg/mL). Briefly the ABTS radical scavenging activity of samples of *R. hastatus* revealed remarkable results with dose dependent response as shown in the Figure 2.

## Discussions

Acetylcholine, an extensively distributed neurotransmitter especially in the central nervous system has a key role in transmission of signal across the synapses, cerebral blood flow, cognitive performance and coordination. The decreased level of acetylcholine within the nervous system of the body may be due to reduced acetyltransferase or increased level of AChE. These two factors are correlated with dementia and AD. The hydrolysis of acetylcholine is decreased by inhibiting AChE in the brain [25,26]. There are numerous evidences which support the deteriorating action of free radicals, which can lead to cognitive ageing and CNS disorders [27]. The age related brain performance can be easily ameliorated by the consumption of fruits and vegetables rich in flavonoids, which possess the ability to neutralize the free radicals [28]. It has already been reported that the cognitive performance can be significantly improved by taking diet rich in antioxidant species especially the flavonoids [29,30]. It is believed that the natural products are safe as compared to synthetic compounds [31]. Due to great interest towards the natural products, the scientists are trying to investigate more and more plants for the presence of effective compounds, which may cure a specific disease. The presence of thousands of flavonoids has been confirmed in more than 3000 plant species. Flavonoids have been reported to be significantly effective in cholinesterase inhibition, i.e., Leufolins A and B (flavonoids) isolated from *Leucas urticifolia* have been scientifically verified to possess strong BChE inhibitory potential [32]. It is also obvious of our current investigations that Rh.Fl possess comparable anticholinesterase potential with the positive control (galantamine). Similarly the saponins are also prominent secondary metabolites, which have been verified to possess various beneficial pharmacological potentials. For instant, the saponins isolated from traditional Chinese medicines have demonstrated excellent antioxidant activity [33]. As obvious of the current investigational data, the Rh.Sp demonstrated good antioxidant and significant anticholinesterase potential. Similarly the antioxidant potential of Rh.Sp against ABTS was comparable to the positive control and Rh.Fl as shown in the Figure 2. The current investigation and the literature review intimates the presence of anticholinesterase compounds in Rh.Sp and Rh.Fl of *R. hastatus* just like the saponins (Bacosides) of *Bacopa monnieri* and the flavonoids (ginkgoflavonglycosides) of Ginkgo biloba [34]. Among the subsequent fractions the excellent activity of Rh.Chf and Rh.EtAc represents that the saponins and flavonoids may be concentrated in the said fractions. The previously reported data also goes parallel with our current investigation i.e., in the current study the Rh.Fl has shown significant antioxidant potential and in the previous reports, the fractions (chloroform, ethyl acetate and butonal) having high quantity of total phenolics and total flavonoids content had shown significant antioxidant activity as reported by Sehreen et al and Afzal et al [35,36]. Likewise several other plant species have been reported to possess multiple pharmacological activities [37-39].

## Conclusions

The thorough literature survey and the results of our current investigation support the potential role of *R. hastatus* in the treatment of nervous disorders. Similarly, based on the significant enzymes (AChE, BChE) inhibition and radicals scavenging demonstrations of various samples of *R. hastatus,* it may be inferred that the Rh. Fl and Rh. Sp of *R. hastatus* may be the best sources of antioxidant and anticholinesterase compounds. The bioguided isolation of *R. hastatus* is in progress in our laboratory, which may lead to new anti-Alzheimer’s disease and anti-ageing candidates.

## Methods

### Plant collection, extraction and fractionation

The whole plant of *R. hastatus* was collected from the hilly area of Gorha Gat nearby University of Malakand, Chakdara (Lower dir) KPK, Pakistan. The plant was identified by plant taxonomist Ali Hazrat and deposited with voucher number (1015SJ) in the herbarium, Department of Botany, Shaheed Benazir Bhutto University, Sheringal (Dir Upper) KPK, Pakistan. All the extra particles (sand and dust) were thoroughly removed from the plant and was scattered on neat paper in a room protected from sunlight and properly shade dried for 20 days. The paper was changed every day to avoid fungal growth. After shade drying the plant material was converted into coarse particles using cutter mill. The powdered plant sample (4 kg approximately) was subjected to maceration in 80% methanol and filtered after 15 days using muslin cloth. The filtrate was concentrated using rotary evaporator and then solidified using water bath at 40°C yielding 300 g of crude methanolic extract. The crude methanolic extract obtained (260 g) was subject to fractionation using successive solvent-solvent extraction process starting from *n*-hexane, chloroform, ethyl acetate and water yielding 30, 22, 53 and 140 g of Rh. Hex, Rh. Chf, Rh. EtAc and Rh. Aq respectively [40].

### Extraction of crude saponins

For the extraction of Rh. Sp from *R. hastatus*, 20 g powdered plant was added to 100mL ethanol (20%). The sample was heated at 55°C in water bath for four hours with continuous stirring. After four hours the sample obtained was filtered and the residue having greenish color was re-extracted with 200 mL of 20% ethanol. The sample after extraction was heated until a concentrated volume of 40 mL was obtained. Then it was transferred into a separating funnel and 20 mL of diethyl ether was added to it. After vigorous shaking, the separating funnel was put in a stand to get two layered sample. The lower layer was collected, which was aqueous layer while the upper diethyl ether layer was discarded. The aqueous layer obtained was diluted with 60 mL of *n*-butanol and the combined *n*-butanol extract was washed with 10 mL of 5% sodium chloride solution. The final solution obtained was kept in a hot water bath until complete evaporation and the Rh. Sp obtained were dried in an oven yielding 1.3 g of Rh. Sp [41].

### Extraction of flavonoids

For the extraction of flavonoids from *R. hastatus*, the procedure of Harborne was followed [42]. Powdered plant sample weighing 20 g was heated at 50°C in 200 mL of 2M HCl under reflux for half an hour. It was cooled and filtered using whatman No.42 filter paper. The filtrate was treated with equal volume of ethyl acetate. The Rh.Fl present in the extract were precipitated, which were recovered with the help of weighed filter paper. The weight of Rh.Fl obtained was 1.6 g (8%) [43].

### Preliminary phytochemical screening

The preliminary phytochemical screening of the *R. hastatus* was carried out to determine the existence of alkaloids, saponins, anthraquinone glycoside, tannins and flavonoids [44].

### Anticholinesterase assays

#### Chemicals required

Electric eel acetylcholinesterase (type-VI-S, Sigma-Aldrich USA), Aquine butyrylcholinesterase (Sigma-Aldrich USA), Acetylthiocholine Iodide (Sigma-Aldrich UK), Butyrylthiocholin iodide (Sigma-Aldrich Switzerland), 5,5-dithio-bis-nitrobenzoic acid (DTNB) (Sigma-Aldrich Germany), Potassium phosphate buffer (pH 8.0), Galantamine from Lycoris Sp. (Sigma-Aldrich France).

#### Spectroscopic analysis

AChE and BChE inhibition was assessed spectrophotometrically for Rh. Fl and Rh. Sp using Acetylthiocholine iodide and Butyrylthiocholine iodide as substrate following the method of Ellman [45]. In this method, 5 μL of AChE (0.03 U/mL) and BChE (0.01 U/mL) were taken in a cuvette and 205 μL of plant samples (125–1000 μg/mL) were added to them. DTNB (5 μL) was added to the mixture, transferred to a water bath having temperature of 30°C and incubated for 15 minutes. Substrates having volume of 5 μL were added to the mixture to start the reaction. The reaction mixture was analyzed at 412 nm using a double beam spectrophotometer. Absorption was recorded for 4 minutes. The formation of yellow color indicated the formation of 5-thio-2-nitrobenzoate anion as a result of the reaction between thiocholines and DTNB. To check the non-enzymatic hydrolysis of substrate, white assay was carried out without enzymes and plant samples. The reaction mixture containing all the components excluding plant sample was taken as control. Percent enzyme activity and percent inhibition were figured out as follows.

$$ \mathrm{V}=\Delta \mathrm{Abs}/\Delta \mathrm{t} $$

$$ \%\ \mathrm{enzyme}\ \mathrm{activity}=\mathrm{V}/\mathrm{Vmax}\times 100 $$

$$ \%\ \mathrm{enzyme}\ \mathrm{inhibition}=100\hbox{-} \%\ \mathrm{enzyme}\ \mathrm{activity} $$

(Where V denotes rate of reaction in the present of inhibitor and Vmax denotes rate of reaction without inhibitor).

### DPPH radical scavenging assay

The method of Brand-Williams et al. [46] with some modifications was followed for the DPPH assay. DPPH (24 mg) was dissolved in 100 mL of methanol to get DPPH solution. The stock solutions of plant samples having concentrations of 1 mg/mL were prepared in methanol and then diluted to the concentrations of 500, 250, 125, 62.5 μg/mL. 0.1 mL diluted solution of each sample was mixed with 3 mL of DPPH solution in methanol. The solution was incubated at 23°C for 30 minutes and then the absorbance was measured at 517 nm. For positive control ascorbic acid was used. Each concentration was taken in triplicate and the data obtained was presented as mean ± S.E.M. The percent radical scavenging activity was calculated using the following equation:

$$ \mathrm{Percent}\ \mathrm{scavenging}\ \mathrm{effect} = \frac{\mathrm{control}\ \mathrm{absorbance}-\mathrm{sample}\ \mathrm{absorbance}}{\mathrm{control}\ \mathrm{absorbance}}\times 100. $$

### Hydrogen peroxide scavenging assay

The hydrogen peroxide scavenging activity of samples was conducted following the procedure of Ruch et al [47]. Hydrogen peroxide solution (2 mM) was prepared in 50 mM phosphate buffer having the pH of 7.4. Various plant samples having volume of 0.1 mL were taken in test tubes and their volumes were made 0.4 mL by addition of 50mM phosphate buffer. Hydrogen peroxide solution (0.6 mL) was added to it and vertexed. After 10 minutes the absorbance of each sample was measured at 230nm against the blank. The hydrogen peroxide scavenging activity was measured using the following equation:

$$ \mathrm{Hydrogen}\ \mathrm{peroxide}\ \mathrm{scavenging}\ \mathrm{activity}=\left(1\hbox{-} \frac{\mathrm{absorbance}\ \mathrm{of}\ \mathrm{sample}}{\mathrm{absorbance}\ \mathrm{of}\ \mathrm{control}}\right)\times 100. $$

### ABTS free radical scavenging assay

Antioxidant potentials of Rh. Sp and Rh. Fl were assessed using the free radicals of 2, 2-azinobis [3-ethylbenzthiazoline]-6-sulfonic acid (ABTS) [48]. Solutions of ABTS 7 mM and potassium persulfate 2.45 mM were prepared and thoroughly mixed. The solution was kept overnight in dark to produce free radicals. After incubation the absorbance of ABTS solution was adjusted to 0.7 at 745 nm by the addition of 50% methanol. Then 300 μl of test samples were taken and 3 mL of ABTS solution was added to it. The solution obtained was transferred to cuvette and absorbance of was measured using a double beam spectrophotometer for six minutes. For positive control Ascorbic acid was used. The data was recorded in triplicate and percent ABTS free radicals scavenging activity was calculated as follows:

$$ \mathrm{Percent}\ \mathrm{scavenging}\ \mathrm{activity} = \frac{\mathrm{control}\ \mathrm{absorbance}-\mathrm{sample}\ \mathrm{absorbance}}{\mathrm{control}\ \mathrm{absorbance}}\times 100. $$

### Estimation of IC_50_ values

Concentrations of test samples, which inhibited substrate hydrolysis (AChE and BChE) by 50% (IC_50_). Radical scavenging activity was determined by a linear regression analysis among the percent inhibition against the test samples concentrations via MS Excel program.

### Statistical data analysis

All the tests were performed in triplicate and values were expressed as means ± S*.*E*.*M. Significance between radical scavenging activity and test samples were analyzed using Mann-Whitney U test. Group comparison was performed by Student’s *t*-test in which the P < 0.05 were considered significant.
